# Aquaporins Are Critical for Provision of Water during Lactation and Intrauterine Progeny Hydration to Maintain Tsetse Fly Reproductive Success

**DOI:** 10.1371/journal.pntd.0002517

**Published:** 2014-04-24

**Authors:** Joshua B. Benoit, Immo A. Hansen, Geoffrey M. Attardo, Veronika Michalková, Paul O. Mireji, Joel L. Bargul, Lisa L. Drake, Daniel K. Masiga, Serap Aksoy

**Affiliations:** 1 Department of Epidemiology of Microbial Diseases, Yale School of Public Health, Yale University, New Haven, Connecticut, United States of America; 2 Department of Biology and Institute of Applied Biosciences, New Mexico State University, Las Cruces, New Mexico, United States of America; 3 Institute of Zoology, Slovak Academy of Sciences, Bratislava, Slovakia; 4 Department of Biochemistry and Molecular Biology, Egerton University, Njoro, Kenya; 5 Molecular Biology and Bioinformatics Unit, International Center of Insect Physiology and Ecology (ICIPE), Nairobi, Kenya; National Institute of Allergy and Infectious Diseases, United States of America

## Abstract

Tsetse flies undergo drastic fluctuations in their water content throughout their adult life history due to events such as blood feeding, dehydration and lactation, an essential feature of the viviparous reproductive biology of tsetse. Aquaporins (AQPs) are transmembrane proteins that allow water and other solutes to permeate through cellular membranes. Here we identify tsetse aquaporin (AQP) genes, examine their expression patterns under different physiological conditions (blood feeding, lactation and stress response) and perform functional analysis of three specific genes utilizing RNA interference (RNAi) gene silencing. Ten putative aquaporins were identified in the *Glossina morsitans morsitans* (*Gmm*) genome, two more than has been previously documented in any other insect. All organs, tissues, and body parts examined had distinct AQP expression patterns. Two AQP genes, *gmmdripa* and *gmmdripb* ( = *gmmaqp1a* and *gmmaqp1b*) are highly expressed in the milk gland/fat body tissues. The whole-body transcript levels of these two genes vary over the course of pregnancy. A set of three AQPs (*gmmaqp5*, *gmmaqp2a*, and *gmmaqp4b*) are expressed highly in the Malpighian tubules. Knockdown of *gmmdripa* and *gmmdripb* reduced the efficiency of water loss following a blood meal, increased dehydration tolerance and reduced heat tolerance of adult females. Knockdown of *gmmdripa* extended pregnancy length, and *gmmdripb* knockdown resulted in extended pregnancy duration and reduced progeny production. We found that knockdown of AQPs increased tsetse milk osmolality and reduced the water content in developing larva. Combined knockdown of *gmmdripa*, *gmmdripb* and *gmmaqp5* extended pregnancy by 4–6 d, reduced pupal production by nearly 50%, increased milk osmolality by 20–25% and led to dehydration of feeding larvae. Based on these results, we conclude that gmmDripA and gmmDripB are critical for diuresis, stress tolerance and intrauterine lactation through the regulation of water and/or other uncharged solutes.

## Author Summary

*Glossina* sp. are responsible for transmission of African trypanosomes, the causative agents of sleeping sickness in humans and Nagana in cattle. Blood feeding and nutrient provisioning through lactation during intrauterine progeny development are periods when considerable water movement occurs within tsetse flies. With the completion of the tsetse fly genome, we sought to characterize the role of aquaporins in relation water homeostasis during blood feeding, stress tolerance and the lactation cycle. We provide evidence that specific AQPs are 1. critical during diuresis following a bloodmeal, 2. important in the regulation of dehydration resistance and heat tolerance and 3. crucial in the allocation of water within tsetse milk that is necessary for progeny hydration. Specifically, we discovered a novel tsetse AQP that is imperative to lactation and may represent a potential target for population control of this disease vector.

## Introduction

Tsetse flies are the major insect vectors of African trypanosome parasites responsible for Human African Trypanosomiasis (HAT)/sleeping sickness and African Animal Trypanosomiasis (AAT)/nagana. AAT has forced farmers and herdsmen to either abandon wide areas of land across Africa or maintain their herd under regular chemotherapy [1]. There are no HAT vaccines and treatment is hampered by the high cost and adverse side effects of drugs [2], [3]. In addition, prevalence of drug resistant trypanosome populations is rising [4]–[6]. Reduction of vector populations therefore remains the cornerstone of trypanosomiasis control. Trapping technologies have been applied to tsetse control with limited success due to socio-economic factors [3], [7]. The development of cheaper and less labor intensive strategies to interrupt tsetse reproduction could be utilized to complement current tsetse and trypanosomiasis control interventions to prevent resurgence of disease similar to what has occurred in the 1990s.

Viviparity (birth of live young) during tsetse reproduction differentiates this fly from insect reproductive systems that utilize oviparous reproduction (deposition of eggs). Tsetse reproductive morphology has undergone significant modifications to carry an offspring throughout larval development. The oviduct has expanded into a uterus to carry an intrauterine larvae [8] and the accessory gland ( = milk gland) is specialized to synthesize and secrete lactation products to feed the developing larvae. The ovaries are reduced in size and capacity to a combined total of four ovarioles [8]. Oogenesis in tsetse begins before eclosion with a single oocyte developing in one ovary. Oocyte development takes 6–7 days to complete. Following completion of oogenesis, the egg is fertilized and ovulated in the uterus [8] where embryonic and larval development occurs. Completion of larvigenesis is followed by parturition of a fully developed third instar larva that pupates within 30 minutes of deposition. Female flies can only produce a maximum of 8–10 offspring in their lifetime due to their slow reproductive rate. This low reproductive output represents a bottleneck that can be utilized as a target to reduce tsetse population.

At parturition each deposited larvae is nearly 6 mm long and weighs 20–25 mg (sometimes more than the mass of the mother). Provision of nutrients to the developing offspring poses a monumental task to tsetse mothers, who will abort gestating offspring without adequate nutrients reserves or access to regular bloodmeals. Nutrients need to be extracted from the bloodmeal, metabolized and stored in the fat body prior to pregnancy to accumulate nutrient stores necessary for lactation [8]–[12]. During pregnancy, nutrients can be acquired directly from bloodmeal digestion and/or from stored nutrients in the fat body for incorporation into milk secretions [8]–[14]. The nutrients are processed by the milk gland, and transferred into the uterus in the form of milk secretions near the larval mouthparts for ingestion. Females produce 20–30 mg (wet weight) of milk during each gonotrophic cycle [13]. The nutrient components of the milk (15–20% of the wet weight) consist of 50% lipids and 50% proteins [13], [15]. Specific protein components of the milk have been identified and include Transferrin [16], multiple Milk Gland Proteins (MGPs) [17]–[19], Sphingomyelinase [20] and Peptidoglycan Recognition Protein (PGRP-LB) [21], [22]. Lipids consist primarily of triacylgylcerols and phospholipids, the majority of which are found as lipid-protein complexes [15]. However, 70–80% of the milk secretion is water and to date no studies have addressed the mechanisms that facilitate the shift of nearly 15–22 mg of water from the hemolymph into the milk gland to act as the solvent for secreted nutrients in the milk.

Aquaporins (AQPs) are transmembrane proteins that allow water and other small solutes to permeate through cellular membranes [23]–[26]. AQP gene numbers vary between different organisms. Thirteen AQP genes have been identified in most mammals, named AQP0-12. Due to a recent genome duplication, the zebrafish has 18 AQP genes, the highest number of all vertebrate model systems analyzed [27]. AQP gene numbers also vary in insects - *Drosophila* and the yellow fever mosquito *Aedes aegypti* have eight and six AQPs, respectively while the malaria mosquito *Anopheles gambiae* has seven [28], [29]. AQPs can be divided into two groups that either transport only water or that transport water, glycerol, urea and other small metabolites [23], [24], [26], [30]. AQPs are critical for diuresis after blood feeding and dehydration tolerance in mosquitoes [28], [29], cold and freezing tolerance in various insects [31]–[35] and promote anhydrobiosis in the African sleeping midge [36], [37]. Previous EST projects on tsetse flies have demonstrated the expression of AQPs in many tissues [38]–[40], but the physiological roles of these channel proteins, particularly in relation to viviparous reproduction and blood feeding, have not been determined.

In this study, we identify the putative tsetse AQP genes, determine their expression in specific female tissues/structures, and analyze transcript levels after blood feeding and throughout pregnancy. In addition, we study the physiological role of specific AQPs during blood feeding, pregnancy and stress exposure by functional knockdowns using RNA interference. Our results show that AQPs are critical for diuresis, environmental stress tolerance, lactation and progeny development in tsetse flies. Interference with the function or expression of these proteins by chemical treatment or other methods could represent a novel tsetse control mechanism with the potential to reduce populations. Finally, we discuss the critical role of AQP as a factor necessary for milk production in both the mammalian and tsetse systems.

## Materials and Methods

### Fly and tissue/structure biological samples

The *Glossina morsitans morsitans* colony maintained at Yale University insectary originated from a small population of flies originally collected in Zimbabwe. Flies are maintained at 24°C and 50–60% RH. Flies receive defibrinated bovine blood via an artificial feeding system every 48 h [41], [42]. Mated female flies were collected for qPCR and western blotting according to established oocyte, embryo and larval developmental markers [17], [18]. Samples utilized to measure the expression of AQPs throughout pregnancy contain only maternal RNA (intrauterine embryo or larva was removed by dissection), were collected 24 h after blood feeding and represents three biological replicates of three-four combined flies. The tissue/structure samples investigated, including the head, salivary glands, midgut, spermatheca, fat body/milk gland, ovary/oocyte, Malpighian tubules, bacteriome, reproductive tract and hindgut, were removed from pregnant female flies (2nd instar larvae present) and 12–24 h after blood feeding. The larvae were also removed during dissections for subsequent analysis. Flies used to assess AQP transcript levels after blood feeding during diuresis were 6–8 d post-eclosion, 48 h since their last bloodmeal and developing intrauterine offspring were removed prior to sample collection.

### AQP sequence and phylogenetic analysis

Full-length coding sequences were acquired by mapping Illumina RNA-Seq reads from Benoit et al. [19] to *G. morsitans* genome scaffolds containing predicted *aqp* genes using CLC Genomics Workbench bioinformatic software (CLC Bio). Genomics scaffolds are available through Vectorbase (www.vectorbase.org). Sequence alignment was performed using the PROMALS3D software package, which considers structural constraints for divergent protein sequences [43], [44] and Clustal [45]. The reconstruction of the evolutionary history of the aquaporin family in dipterans was performed using Mega 5 [46]. Phylogenetic analysis using neighbor-joining, maximum likelihood, maximum parsimony, as well as Bayesian methods produced similar tree topologies. NPA domains were identified after amino acid sequence alignment, and transmembrane domains were predicted by CLC Workbench (CLC Bio) and the TMHMM Server v. 2.0 (www.cbs.dtu.dk). The most closely related *D. melanogaster* homolog was identified through tBLASTx at Flybase (www.flybase.org).

### Quantitative PCR

Gene-specific primers were developed using the CLC Main workbench software (Table S1). Total RNA was obtained from adult females with Trizol reagent according to manufacturer's protocol (Invitrogen). Tissue/structure-specific RNAs were isolated after dissection from five or six flies. Transcript levels were determined by quantitative RT-PCR (qPCR) with a CFX96 PCR detection system (Bio-Rad, Hercules, CA). Samples were collected in triplicate and normalized to tsetse *tubulin* (*gmmtub*, DQ377071.1) and analyzed with CFX Manager software version 3.1 (Bio-Rad).

### *In situ* hybridization and immunohistochemistry

Milk gland tubules were collected from mated female flies with third instar larvae and placed directly into Carnoy's fixative for a five day fixation period [47]. Samples were prepared according to Attardo et al. [47] using Digoxigenin-labeled RNA probes generated using the MAXIscript T7 transcription kit following manufacturer's protocol (Ambion, Austin, TX) using a primer set with a T7 reverse primer (Table S1). Antibody solutions were made featuring anti-Digoxigenin-rhodamine Fab fragments for FISH probe detection (1∶200 dilution) (Roche) and rabbit anti-gmmMGP (1∶2500) antibodies [17], [47]. Alexa Fluor 488 goat anti-rabbit IgG (Invitrogen) at a dilution of 1∶500 was added as a secondary antibody for immunohistochemistry [47]. Slides were mounted using VECTA SHIELD Mounting Medium with DAPI (Vector Laboratories, Burlingame, CA). Samples were observed using a Zeiss Axioskop2 microscope (Zeiss, Thornwood, NY) equipped with a fluorescent filter. Samples were viewed and imaged at 400× magnification. Images were captured using an Infinity1 USB 2.0 camera and software (Lumenera Corporation, Ottawa, Ontario, Canada).

### RNAi-mediated knockdown of specific AQPs

RNA interference techniques were previously developed for gene knockdown in pregnant tsetse flies [9], [10]. pCRII-TOPO plasmid containing cDNA clones for *gmmdripa*, *gmmdripb* and *gmmaqp5* and a plasmid containing *gfp* (control) served as templates for PCR amplification. The T7 promoter sequence was added to the 5′ end of the primer sequences and PCR amplification conditions are described (Table S1). The PCR products were purified using QIAquick PCR purification kit (Qiagen, Valencia, CA) and cloned into pGEM T-Easy vector (Promega, Madison, WI) and verified by sequencing (Keck DNA sequencing facility, Yale University). dsRNAs were synthesized using the MEGAscript RNAi Kit (Ambion, Austin, TX), purified using a RNeasy Mini Kit (Qiagen, Valencia, CA) and siRNAs were generated by using the Block-iT Dicer RNAi kit (Invitrogen, Carlsbad, CA). The siRNA concentration was adjusted to 600–800 ng/µl in PBS and each fly was injected with 1.5 µl siRNA using a pulled glass capillary needle into their thorax. For combined injection targeting *gmmdripa*, *gmmdripb* and *gmmaqp5*, concentration of each siRNAs were kept at 600–800 ng/µl within the 1.5 µl injected (1800–2400 ng/µl combined). Expression levels of AQP transcripts were determined by qPCR and normalized to *tubulin* to validate transcript suppression. In addition, transcript expression was measured by qPCR for three antioxidant enzymes (Mn/Fe superoxide dismutase; Cu/Zn superoxide dismutase; catalase) normalized to *tubulin* was determined 4–6 h after blood feeding following knockdown of AQPs.

### Characterization of phenotypic traits after AQP RNAi

Multiple phenotypic traits were measured following AQP knockdown. Diuresis/post-blood feeding water loss assays were conducted according to those developed for *Ae. aegypti* with some modifications [28]. Three-four days after siRNA injection of five day old female flies, individuals received a blood meal and were allowed 10 minutes to rest at colony conditions (Figure S1). Subsequently, flies were weighed and moved to 0% RH at 25°C to ensure that any mass changes reflect loss with no interference from atmospheric humidity [48], [49]. Flies were reweighed at 30 minute intervals for 8 h. After 8 h, flies were moved to 0% RH at 65°C, and held until the mass was constant (dry mass) to determine water mass at each weighing interval (water mass at each weighing interval = total mass at each interval - dry mass). The net water loss rate was determined by the slope of a regression according to established methods [44], [45], where ln (m_t_/m_0_) is plotted versus the time based on the exponential model (m_t_ = m_0_ e^−kt^). For this model, m_t_ denotes the water mass at any time t, m_0_ is the initial water mass and k is the rate in %/h. In addition, dehydration/starvation tolerance was assessed based on techniques previously developed for adult flies [48]. Briefly, flies were injected with different siRNAs 3–5 d after emergence and fed 1 d after siRNA injection, moved to colony conditions (24°C and 50–60% RH) and provided no further blood meals. Fly survival was monitored at 12 hour intervals for each group.

Fecundity changes following suppression of AQPs were measured by determining the length of each gonotrophic cycle and the number of progeny produced per female according to Benoit et al. [9]. Flies were injected with siRNA 8–10 d after adult emergence before the increase in AQP transcript expression that occurs during the early portion of larvigenesis before lactation-associated genes are expressed at high levels [19]. The length of the gonotrophic cycle was determined as the day of the first pupal deposition. The progeny produced per female was determined over 40 d, which encompasses the first and second gonotrophic cycles.

Heat tolerance was assessed according to protocols developed for establishing heat tolerance of flies [50]. Female flies were injected with siRNA 3–5 d after adult emergence. Flies were moved to either 40°C, 43.5°C or 47°C for 2 h and subsequently returned to normal colony conditions (24°C and 50–60% RH). Fly survival was measured 24 h post-heat exposure by response to mechanical disturbance with a glass probe.

Changes in the tsetse milk osmolality after knockdown were assessed. Third instar larvae were surgically removed from siRNA-treated pregnant females (injected with siRNA 8–10 d after emergence as before) and their digestive tract was removed. A pulled glass capillary needle was used to recover content from the larval digestive tract using reverse pressure. Contents were combined from three larvae and the osmolality was determined with a Wescor osmometer (Vapro 5600, Logan, Utah, USA).

Larval water content was determined according to protocols developed to assess water content within dipteran larvae [51]. Larvae were removed from the uterus of the mother by dissection. Dissected larvae were blotted dry with paper towels, weighed and moved to 0% RH, 65°C until the mass remained constant ( = dry mass). Water mass was determined as before and the percent water content within the flies was determined by water mass/(water mass+dry mass).

### Statistical analysis

Results in this study were compared utilizing JMP or SAS statistical software programs (Cary, North Carolina, USA). Mean differences utilized between treatments were compared with ANOVA with a Bonferroni correction followed by Tukey's post-hoc test. Survival results were analyzed using a Kaplan-Meier plot with a log rank test.

## Results

### Genomic analysis revealed ten *G. morsitans* AQP genes

Ten putative AQP genes were recovered from the *G. m. morsitans* genome, cDNA libraries from RNA-seq projects [19] and previous EST projects [38], [39]. Comparison of AQP genes with those from other flies revealed that *G. m. morsitans* has two more AQP genes than *D. melanogaster* and four more than *A. aegypti* [28]. All of the AQP genes were located on unique genomic scaffolds with the exception of *gmmaqp4b*/*gmmaqp5* and *gmmaqp4a*/*gmmaqp4c*, which are on two separate scaffolds, respectively (Table 1). Seven of the *Glossina* AQPs carry the NPA (asparagine-proline-alanine)-NPA motif that is typically associated with the AQP family of proteins (Table 1; Figure S2 [52], [53]). Two AQPs (gmmAQP4a and gmmAQP5) have slight variations in this motif, but these changes are common in many organisms [52], [53]. GmmAQP6 has the most modified motif consisting of a CPY-NPV motif that is also documented in *Drosophila*, mosquitoes and *Apis mellifera* [52]. GmmAQP6 is likely a member of an insect specific aquaporin [52], [53]. *Glossina* AQPs contain the characteristic 6 transmembrane domains with the exceptions of gmmAQP4c (5 domains) and the gmmAQP6 (4 domains) (Table 1).

**Table 1:** Sequence information for *Glossina* aquaporins.

Gene	Gene ID	Genomic scaffold	Motif	Transmembrane domains	*Drosophila* homolog
*aquaporin 2a*	*gmmaqp2a* (JN685583)	scf7180000642870	NPA-NPA	6	CG7777 (NP_725052.1)
		scf7180000648444			
*aquaporin 2b*	*gmmaqp2b* (JN685584)	scf7180000641357	NPA-NPA	6	CG7777 (NP_725052.1)
*aquaporin 4a*	*gmmaqp4a* (JN685585)	scf7180000648879	NPV-NPA	6	CG4019 (NP_611813.1)
*aquaporin 4b*	*gmmaqp4b* (JN685586)	scf7180000651845	NPA-NPA	6	CG17664 (NP_611611.3)
*aquaporin 4c*	*gmmaqp4c* (JN685587)	scf7180000648879	NPA-NPA	5	CG17664 (NP_611611.3)
*aquaporin 5*	*gmmaqp5* (JN685588)	scf7180000651845	NPM-NPA	6	CG4019 (NP_611813.1)
*aquaporin 6*	*gmmaqp6* (JN685589)	scf7180000648776	CPY-NPV	4	CG12251 (NP_523728.1)
*big brain*	*gmmbib* (*gmmaqp3*, JN685590)	scf7180000652158	NPA-NPA	6	CG4722 (NP_476837)
*Drosophila integral protein A*	*gmmDripA* (*gmmaqp1a*, JN685581)	scf7180000645389	NPA-NPA	6	CG9023 (NP_523697.1)
*Drosophila integral protein B*	*gmmDripB* (*gmmaqp1*, JN685582)	scf7180000650443	NPA-NPA	6	CG9023 (NP_523697.1)
		scf7180000644745			

**Figure 1:**
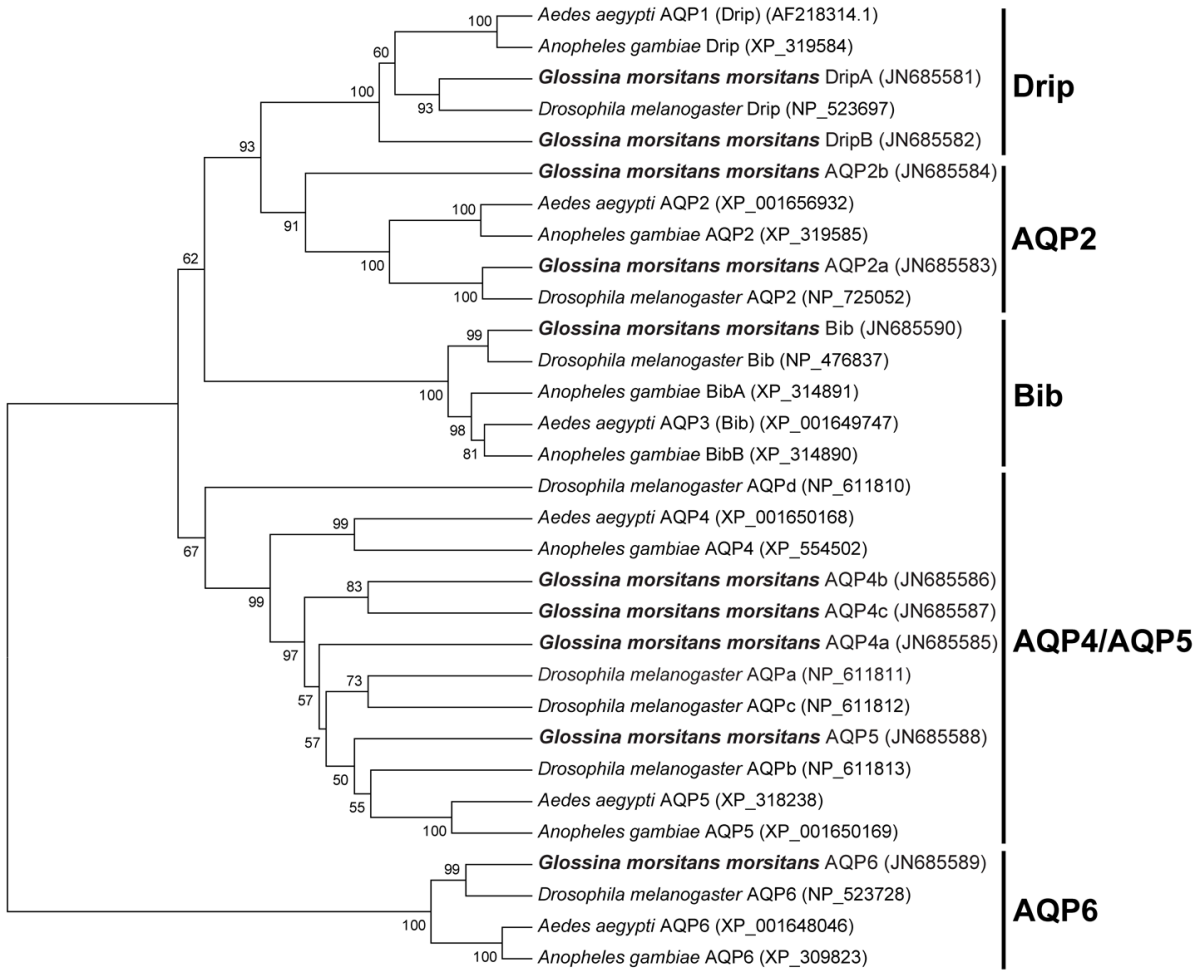
Phylogenetic analysis of the tsetse (*Glossina m. morsitans*) aquaporins in comparison to those from other dipterans. The bootstrap consensus tree was inferred from 10000 replicates and represents the evolutionary history of the taxa examined. This analysis involves 31 sequences. Initial sequence alignment was completed using PROMALS3D server (PROfile Multiple Alignment with predicted Local Structures and 3D constraints) according to Pei et al. [43], [44] and Clustal according to Larkin et al. [45]. Evolutionary analyses and tree generation were conducted in MEGA5 [46].

Our phylogenetic analysis showed that tsetse flies have retained the AQP genes present in *Drosophila* and other dipterans. The major difference is that the *Glossina* genome contains two genes encoding AQPs homologous to *Drosophila drip* (53.8% identity, Figure S2) and two genes homologues to *Drosophila aqp2* (44.3% identity, Figure S2) while all other dipteran genomes currently available contain only one of each. We named these genes *gmmdripa*, *gmmdripb* and *gmmaqp2a*, *gmmaqp2b* respectively (Figure 1). In contrast, the expansion of the AQP4 gene seems to be specific for flies from the suborder Brachycera and does not occur in the two mosquito species (suborder nematocera). We named these three tsetse genes *gmmaqp4a*, *gmmaqp4b*, and *gmmaqp4c*. In *Glossina* these genes share 39–40% identity with each other.

### Three AQP genes are differentially expressed throughout tsetse pregnancy

**Figure 2:**
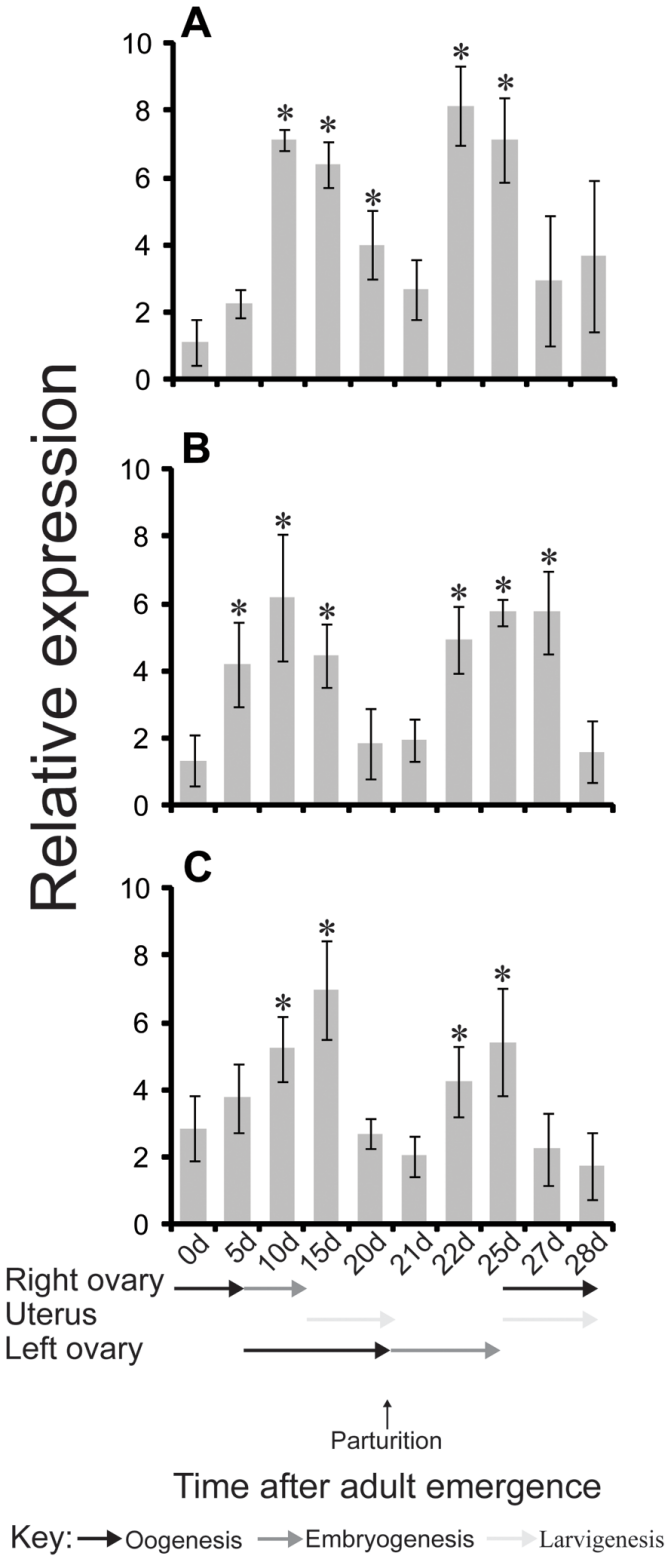
Expression of *gmmdripa*, *gmmdripb* and *gmmaqp5* transcripts during tsetse pregnancy. (A) *gmmdripa*, (B) *gmmdripb* and (C) *gmmaqp5*. Transcript levels were determined by qRT-PCR analysis. The data were analyzed with software version 3.1 (Bio-Rad). Data represent the mean ± SE for four samples and was normalized to *tubulin*. * indicates that the expression is significantly higher (P<0.05) than in newly emerged teneral flies (0 d).

When AQP transcript levels were assessed throughout pregnancy, three AQPs were found to vary significantly over the course of pregnancy (Figure 2, Table S2). The transcript levels for *gmmdripa*, *gmmdripb* and *gmmaqp5* increased immediately prior to larvigenesis, declined during lactation/involution and then increased again 48 h post-parturition before the second round of larvigenesis (Figure 2). These results suggest that these three AQPs may be particularly involved in maintaining water levels throughout tsetse pregnancy.

**Figure 3:**
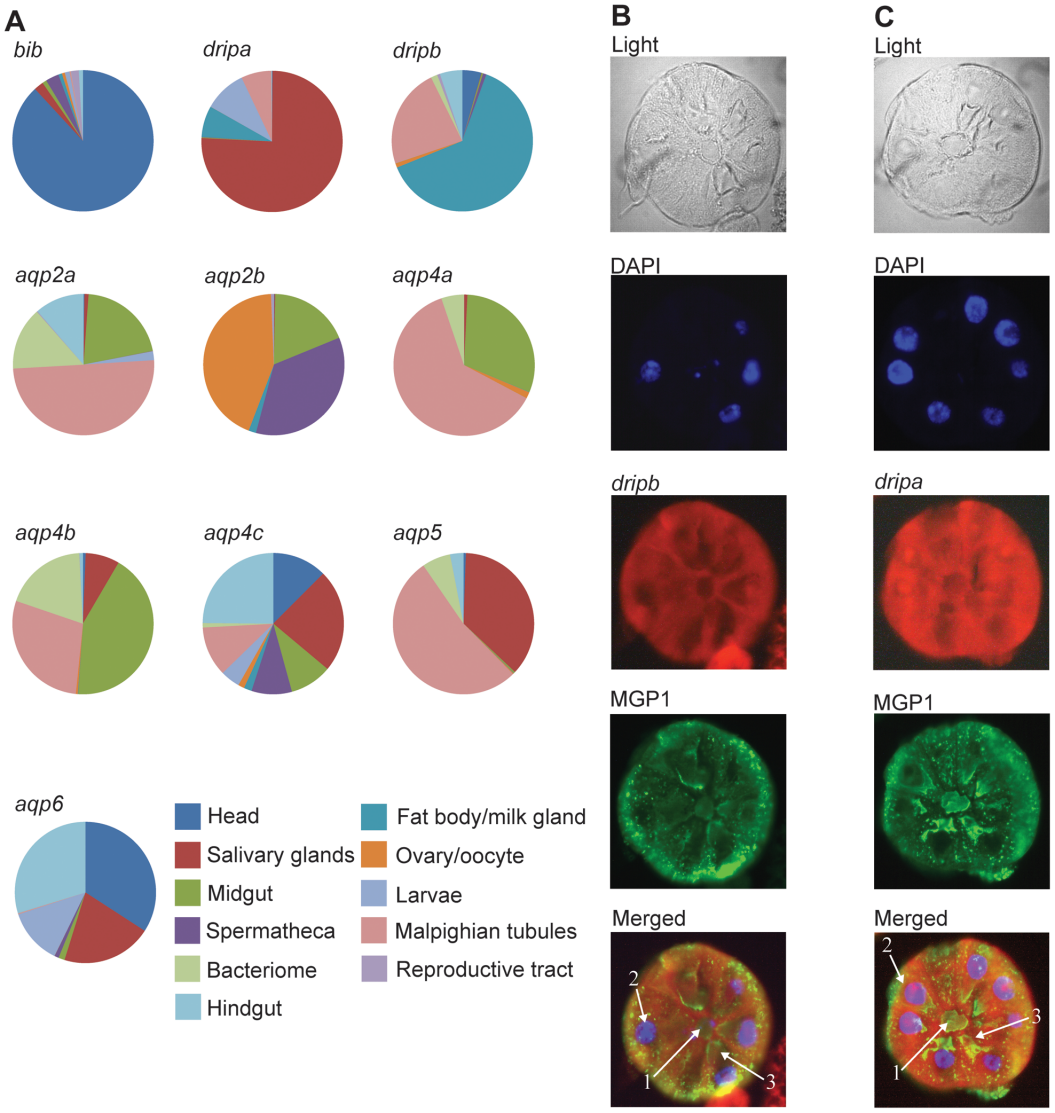
Spatial analysis of AQP transcripts in different tsetse tissues/structures. (A) Relative expression levels of each AQP gene within specific tissues/structures based on qPCR analysis. (B) *gmmdripb* and (C) *gmmdripa in situ* hybridization, red, along with milk gland protein (MGP) immunohistochemistry, green, and DAPI staining of nuclei, blue, of a cross section of milk gland tubules. 1 = milk gland lumen; 2 = nuclei; 3 = secretory reservoir. Negative controls not treated with Digoxigenin-labeled sense RNA probes displayed no signal (Figure S3).

AQP gene expression levels in tsetse females vary significantly between the 11 different tissues and structures analyzed, including the head, salivary glands, midgut, spermatheca, fat body/milk gland, ovary/oocyte, Malpighian tubules, bacteriome, reproductive tract and hindgut (Figure 3). The spatial expression profile and transcript levels of *aqp* genes were compared relative to the average *aqp* expression value per tissue or structure. The expression levels of *gmmaqp5*, *gmmaqp4a*, *gmmaqp4b*, *gmmaqp2a*, *gmmdripa* and *gmmdripb* were significantly higher in the Malpighian tubules than that detected in all other tissues/structures analyzed (P<0.05), and the expression of *gmmaqp2b*, *gmmaqp4a*, *gmmaqp2a* and *gmmaqp4b* were elevated in the midgut (P<0.05, Figure 3a; Table S3). Levels of *gmmaqp2a*, *gmmaqp4a*, *gmmaqp4b* and *gmmaqp5* were also elevated in the bacteriome organ (P<0.05; Figure 3a; Table S2). Within the fat body/milk gland samples, only *dripa* and *dripb* were expressed at high levels compared to other tissues and structures sampled (P<0.05). In addition, we utilized *in situ* hybridization to validate the expression of *gmmdripa*, *gmmdripb* and *gmmaqp5* (genes found to change in expression throughout pregnancy; Figure 2) in the milk gland tubules (Figure 3b–d). This localization verified that particularly the *gmmdripa* and *gmmdripb* genes are expressed in the milk gland tubules that are actively secreting milk products, denoted by the green secretory vesicles in the milk gland cell cytoplasm (Figure 3b–d). Based on these results, AQPs have a distinct spatial-specific expression profile in tsetse with multiple AQPs expressed preferentially in the Malphigian tubules, midgut and milk gland/fat body. Specifically, transcripts for *gmmdripa* and *gmmdripb* are present at high levels in the milk gland organ relative to *gmmaqp5*, which is expressed at much lower levels. The expression profile of the tsetse AQP genes differed following blood feeding (Table 2; Table S4). All tsetse AQP genes increased in expression during at least one time point 6–48 h after a bloodmeal with the exception of *gmmaqp6* (Table 2; Table S4). The genes encoding gmmDripA, gmmDripB, gmmAQP2a, gmmAQP2b and gmmAQP4a each had higher transcript levels at multiple time points after blood feeding (Table 2; Table S4). The AQP transcript levels declined to constitutive levels within 72 h after a blood meal (Table 2; Table S4). These results indicate that multiple tsetse AQPs display increased transcript levels during the blood digestion and diuresis processes after a bloodmeal.

**Table 2:** AQPs that were significantly higher than the temporal average at specific time points after blood feeding based on results from Table S3.

	Summary of transcript highly expressed after blood feeding:
Increased at 0 hour	None
Increased at 2 hour	None
Increased at 6 hour	*gmmdripb, gmmaqp2a, gmmaqp2b, gmmaqp4c, gmmaqp5*
Increased at 12 hour	*gmmbib, gmmdripa, gmmdripb, gmmaqp2a, gmmaqp2b, gmmaqp4a*
Increased at 24 hour	*gmmdripa, gmmdripb, gmmaqp2a, gmmaqp2b, gmmaqp4a, gmmaqp4b*
Increased at 48 hour	*gmmdripa, gmmdripb, gmmaqp2a, gmmaqp2b*
Increased at 72 hour	None
Increased at 120 hour	None

Statistical differences were tested by ANOVA following Bonferroni correction (P<0.05).

### AQP knockdown impaired blood feeding, stress tolerance and milk production

**Figure 4:**
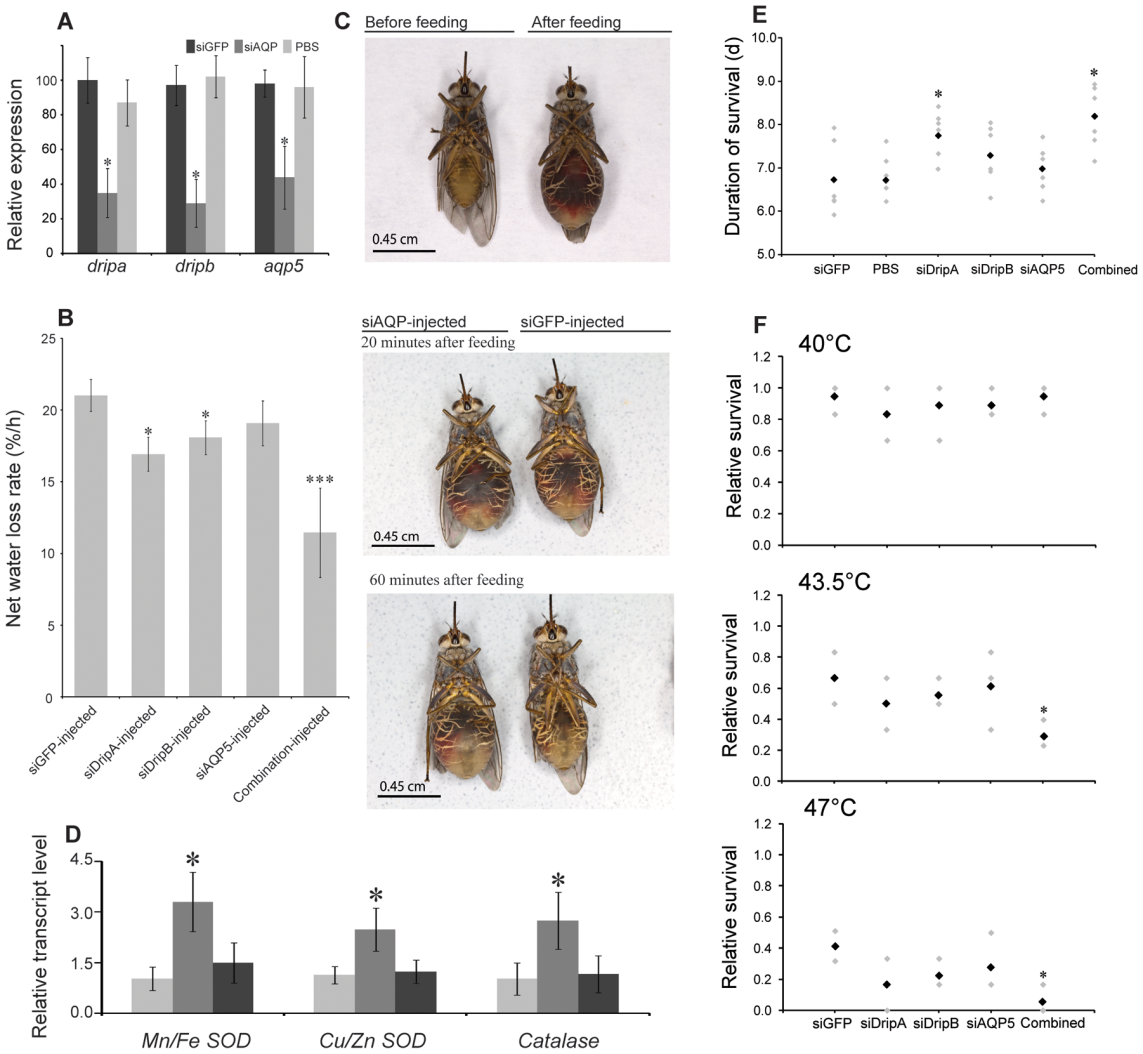
Changes in tsetse physiology after suppression of aquaporins. (A) qPCR expression of *aquaporins* (*gmmdripa*, *gmmdripb* and *gmmaqp5*) after knockdown utilizing siRNA injection. Data represent the mean ± SE for three samples and was normalized to *tubulin*. (B) Rate of water loss (%/h, diuresis plus cuticular and respiratory water loss) after siRNA injection and a subsequent bloodmeal. The combined injection group received all three siRNAs for *dripa*, *dripb* and *aqp5*. Data represent the mean ± SE of three groups of 6 flies. (C) Images of bloodfed flies after injection of siGFP or combined siAQPs showing increased size due to delayed water loss (diuresis along with cuticular and respiratory water loss). (D) qPCR expression of transcripts for antioxidant enzymes (*Mn/Fe superoxide dismutase*, *Mn/Fe sod*; *Cu/Zn superoxide dismutase*, *Cu/Zn sod*; *catalase*, *cat*) 6 h after blood feeding after combined knockdown of AQPs. Data represent the mean ± SE for three samples and was normalized to *tubulin*. (E) Average duration of survival under dehydrating conditions following blood feeding after suppression by siRNA injection. Each point represents mean ± SE of five groups of 10 flies. Black point represents the mean. (F) Heat tolerance following knockdown of *aqps*. n Data are presented as mean ± SE of three groups of 6 flies. Black point represents the mean. * indicates that the value is significantly different (*P<0.05; **, P<0.01; ***, P<0.001) than control. siGFP, short-interfering green fluorescent protein that serves as a control.

We evaluated the functional roles of the putative gmmDripA, gmmDripB and gmmAQP5 proteins, since these genes displayed differential expression throughout tsetse pregnancy. Furthermore, we have previously shown that other genes, such as *brummer lipase* and *methoprene tolerant*, with a similar expression profile are critical to lactation even if they are not expressed in the milk gland [10], [43]. We used an RNAi knockdown approach to understand the roles of these putative proteins during diuresis, blood engorgement, dehydration/starvation, heat tolerance, fecundity and milk osmolality. The qPCR analysis determined that the siRNA treatments significantly reduced transcript levels for these three AQPs by at least 60–70% (Figure 4a). Suppression of *gmmdripa* and *gmmdripb* extended diuresis (Figure 4b,c). These flies were engorged for much longer than the control siGFP treated flies (Figure 4b,c). Combined knockdown through injection of siRNA for *gmmdripa*, *gmmdripb* and *gmmaqp5* had a more severe phenotype showing further reduction in the rate of diuresis/water loss after blood feeding than that observed in individual knockdowns (Figure 4b). We examined the expression of three antioxidant enzyme genes to determine if the delayed water loss after blood feeding could result in increased osmotic stress since overhydration that has been shown to result in oxidative stress [54]. We found that in addition to extending diuresis and engorgement, AQP knockdown increased transcript levels of the antioxidant enzymes (AOEs), *Cu/Zn superoxide dismutase* (*sod*), *Mn/Fe sod* and *catalase*, 4 h post blood feeding in comparison to control siGFP treated flies (Figure 4d). These results suggest that multiple AQP proteins, including gmmDripA and gmmDripB, but not gmmAQP5, play a role during diuresis. Delayed diuresis appears to increase oxidative stress, resulting in increased transcript expression of AOEs.

We next evaluated the impact of individual knockdowns upon dehydration/starvation resistance and heat tolerance. Knockdown of *gmmdripa* improved the ability of flies to survive starvation following a bloodmeal by nearly 0.7 d in comparison to controls (Figure 4e). This effect was further extended to over 1.2 d when multiple AQP genes were knocked down (Figure 4e). In contrast to the starvation/dehydration resistance, heat tolerance of flies was impaired by suppression of AQPs (Figure 4f). Knockdown of *gmmdripa*, *gmmdripb* and *gmmaqp5* individually did not lower tsetse's heat tolerance significantly. However, a combined reduction of all three genes impaired tsetse's heat tolerance by 40–50% and increased the ability of flies to tolerate starvation/dehydration and decreased their ability to endure thermal stress.

**Figure 5:**
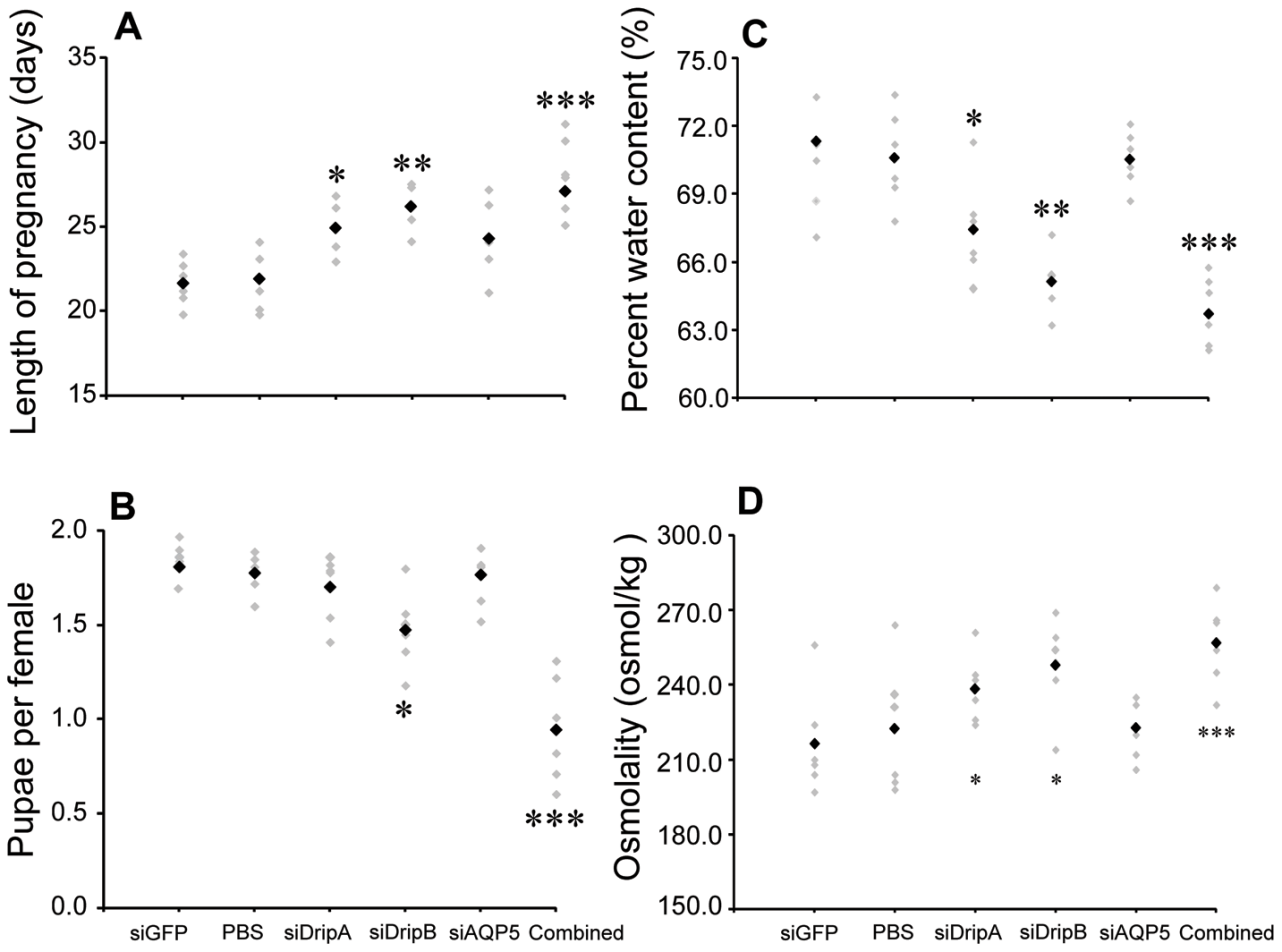
Fecundity, gonotrophic cycle duration, milk osmolality and percent water content of intrauterine larva following knockdown of *aquaporins* in the mother. (A) Length of 1st gonotrophic cycle. Black point represents the mean. (B) Average pupae produced per female over 40 d after suppression of *gmmdripa*, *gmmdripb*, and *gmmaqp5* by siRNA injection. For “combined injection”, flies were simultaneously injected with *gmmdripa*, *gmmdripb* and *gmmaqp5* siRNA. Data are presented as mean ± SE of three groups of 20 flies. Black point represents the mean. (C) Percent water content in intrauterine larvae after suppression of gmm*dripa*, *gmmdripb*, *gmmaqp5*, *gfp* by siRNA injection. Data are presented as mean ± SE of three groups of 15 flies. Black point represents the mean. (D) Osmolality of tsetse milk recovered from larval gut contents after suppression of *gmmdripa*, *gmmdripb*, *gmmaqp5* and *gfp* by siRNA injection. Data are presented as mean ± SE of three groups of 6 flies. Black point represents the mean. * indicates that the value is significantly different (*, P<0.05; **, P<0.01; ***, P<0.001) than control. siGFP, short-interfering Green Fluorescent Protein was used for internal control. Black point represents the mean.

In relation to fecundity, knockdown of *gmmdripa* resulted in a delay in pregnancy, although pupae produced by each female per gonotrophic cycle was not significantly reduced (Figure 5a,b). Suppression of *gmmdripb* led to a significant prolonged pregnancy and reduced fecundity (Figure 5a,b), while suppression of *gmmaqp5* did not impact either. Combined inhibition of all three AQPs led to the most drastic impact on fecundity, resulting in a 6 day (30%) extension in the duration of pregnancy and ∼50% reduction in pupae deposition number.

We also observed that the water content in the developing larvae and osmolality of milk secretions was altered after AQP knockdown (Figure 5c,d). Reduction of only *gmmdripa* or *gmmdripb* yielded milk secretion with a 20–25% increased osmolality when analyzed from 3rd instar larvae guts in comparison to control flies (Figure 5d). When combined *gmmdripa*, *gmmdripb* and *gmmaqp5* reduction was analyzed, milk osmolality was further increased, albeit only slightly more than individual knockdown of *gmmdripa* or *gmmdripb*, respectively (Figure 5d). Reduction of *gmmdripa* and *gmmdripb* also caused only a 3–4% and 5–6% reduction in larval water content, respectively. Simultaneous suppression of all three AQPs resulted in a 7–9% reduction in larval water content (Figure 5c). Thus, AQP knockdown leads to increased osmolality of the milk due to reduced water levels that likely promotes larval dehydration.

To summarize, our results show that AQPs play a critical role in multiple aspects of tsetse's physiology. Tsetse AQPs are critical to the maintenance of water balance (since knockdown impairs diuresis and increases dehydration tolerance). Tsetse heat tolerance is impaired upon AQP knockdown. Lastly, multiple AQPs contribute to tsetse's fecundity, with both gmmDripA and gmmDripB playing the most critical role during pregnancy.

## Discussion

In this study, we identified ten genes coding for putative AQPs and examined their role(s) during water homeostasis and reproduction in tsetse flies. Transcript levels of multiple AQPs varied throughout pregnancy, during diuresis and in relation to blood digestion after feeding. After blood feeding, all tsetse AQP transcript levels were significantly higher than unfed levels for at least one of the collection time points tested with the exception of *gmmaqp6*. This suggests that AQPs likely play a role for the osmotic changes associated with blood ingestion, which necessitate significant amount of water movement while the flies undergo diuresis to reach pre-feeding water levels. Three AQPs varied in expression throughout pregnancy, suggesting that specific AQPs may be critical during lactation and/or intrauterine larval development. Individual knockdown of *gmmdripa* and *gmmdripb* reduced the rate of diuresis after blood feeding, increased dehydration tolerance, impaired heat tolerance and delayed larval development. Suppression of *gmmdripb* had the most detrimental effects on reproduction, resulting in a drastic extension in pregnancy duration, substantial changes in milk osmolality, reduction in fecundity and reduced water content in the intrauterine larvae. Combined knockdown of multiple AQPs exacerbated the negative effect of individual knockdowns, leading to ∼50% reduction in fecundity. These results indicate that AQPs are critical for thermal tolerance, dehydration/starvation resistance, diuresis and fecundity in tsetse females.

### Expansion of the *Glossina* AQP family in relation to other flies

Information on insect aquaporin functions have expanded as studies have been conducted on multiple species under different physiological conditions [26]. Current knowledge indicates that most insect species have 5–8 AQPs that are divided into at least six distinct subfamilies [26], [28]. Within Diptera, lower flies (Nematocera) typically have 5–6 AQP genes and higher flies (Brachycera) usually have 1–2 more [26], [28]. Interestingly, tsetse flies have the highest number of AQPs documented for any insect with 10 distinct genes. The additional two AQP genes in *G. morsitans* are closely related to the *Drosophila drip* and *aqp2* genes. Given the expanded number of aquaporin genes present in tsetse, we investigated their role under different physiological states, particularly during diuresis and tsetse's viviparous reproductive biology

### AQPs are critical for tsetse diuresis and dehydration

The role AQPs play during diuresis has been investigated in multiple insects. In the yellow fever mosquito *A. aegypti*, knockdown of individual AQPs had differential effect on the rate of diuresis. The reduction of diuresis after a single AQP knockdown was exacerbated when multiple genes were suppressed in unison [28]. For tsetse flies, we show that diuresis is impaired by individual suppression of *gmmdripa* and *gmmdripb*, but not *gmmaqp5*. Combined knockdown of the three AQPs led to a 50% reduction in the rate of diuresis. These results indicate that multiple tsetse AQPs are critical to diuresis and may be partially redundant in function. Along with these results, we demonstrate that the delay in diuresis leads to increased expression of AOEs. This increase in AOEs is likely due to extended periods of excess water content following blood feeding since it has been demonstrated that overhydration can result in oxidative stress and increased expression of AOEs in the Antarctic midge, *Belgica antarctica* [55]. The results of our AQP knockdown experiments stress the importance of AQPs for diuresis and feeding-induced water stress in tsetse flies.

Dehydration tolerance is critical for the survival of insects when water is not readily available and the relative humidity is low [56]. Studies on the malaria mosquito, *An. gambiae*, showed that knockdown of AQP1 leads to extended survival under dehydrating conditions [29]. In *B. antarctica*, suppression of AQPs using HgCl_2_ increased cellular dehydration tolerance [35]. In tsetse, reduction of *gmmdripa* enhanced the dehydration tolerance and simultaneous knockdown of *gmmdripa*, *gmmdripb* and *gmmaqp5* increased dehydration/starvation tolerance even further. The increase in dehydration/starvation tolerance after AQP knockdown is likely the result of reduced water loss through the Malpighian tubules [29]. These results suggest that reduced AQP levels could be a general mechanism by which insects increase dehydration resistance.

### Heat tolerance is impaired by AQP suppression

Many studies have addressed the role of AQPs during insect cold tolerance. In *B. antarctica*, the gall fly, *Eurosta solidaginis*, and the rice stem borer, *Chilo suppressalis*, functional AQPs are critical for cold tolerance [31], [32], [34], [35]. Our study is the first to address the role of AQPs in relation to heat tolerance. Heat tolerance of tsetse is significantly impaired following knockdown of AQPs. As dehydration tolerance is improved, the rate of water loss is slightly lower after knockdown of AQPs (this study [28], [29]). We hypothesize that due to this effect, evaporative cooling that typically occurs in tsetse with rising temperature may be reduced, leading to impaired heat tolerance. This evaporative cooling may occur via water loss directly through the cuticle or through the evaporation of water when spiracles are open during respiration [57], [58]. In addition to evaporative cooling through cuticular or respiratory water loss, diuresis after blood feeding was shown to substantially improve heat tolerance in mosquitoes [59]. A similar mechanism of reducing body temperature through diuresis may operate in tsetse flies under periods of heat stress. Further studies will be necessary to determine if evaporative cooling occurs while tsetse is actively blood feeding.

### Two *Glossina* AQPs provide water necessary for tsetse lactation

One of the major goals of this study was to identify the AQPs that may be critical during periods of tsetse lactation. We found that two AQPs, *gmmdripa* and *gmmdripb*, are expressed in the fat body/milk gland structure (Figure 3a). In addition, three AQPs (*gmmdripa*, *gmmdripb* and *gmmaqp5*) showed differential expression throughout tsetse fly pregnancy (Figure 3). Based on their transcript localization and expression during pregnancy, we focused on the role of *gmmdripa*, *gmmdripb* and *gmmaqp5* in relation to tsetse lactation. Knockdown of both *gmmdripa* and *gmmdripb* individually resulted in an extension of pregnancy duration. Osmolality of the milk and water content within the larvae after knockdown of *gmmaqp5* was not affected, indicating this AQP is not likely critical during tsetse lactation. The phenotypic gonotrophic delay, reduced fecundity (only for *gmmdripb*) and lower larval water content after knockdown coupled with the high expression of *dripa* and *dripb* in the milk gland, suggests that these two water channel proteins are critical for supplying the water required to generate milk. gmmAQP5 likely does not have a major role during tsetse lactation, rather it may play a role in the Malpighian tubules or salivary glands that may results in its varying expression throughout tsetse pregnancy. In addition, AQP5 has been described to be multi-functional with the ability to transport other molecules such as urea and polyols such as glycerol [60], [61]. This suggests that gmmAQP5 might also transport molecules other than water within the tissues where it is expressed (Malpighian tubules, salivary glands, bacteriome and/or hindgut) which may be critical to tsetse's physiology. Based on our results we suggest that pharmacological interference with AQPs could significantly reduce tsetse fly fecundity and represent a potential novel strategy for tsetse control.

### Potential role of AQPs in relation to HAT and AAT

It remains to be seen whether AQPs may influence tsetse's trypanosome transmission ability. The localization of specific AQP expression to the salivary glands and midgut tissues suggests that AQPs may be involved in the interactions between tsetse and trypanosomes ni these locations. Tsetse AQPs likely have two potential roles that could directly aid or impede trypanosome infection: 1. AQPs could serve as a site for cell adhesion due to their presence on the surface of cells, particularly in the salivary glands or 2. regulation of osmolality within locations where parasites reside. Although these two possibilities must be investigated, changes in osmolarity and electrolyte levels have been shown to be critical to development and survival of *Trypansoma cruzi* in kissing bugs [62], [63]. Recently within parasite infected salivary glands, we identified that transcript levels of *Drosophila* integral protein A (DripA) is slightly increased [64], suggesting that this AQP may have a role in host-parasite interactions in the salivary glands.

### AQPs: Critical for water provisioning during tsetse and mammalian lactation

Our recent studies indicate that tsetse milk production is partially analogous in function to lactation in mammalian systems [10], [16], [20]. These similarities include the presence of specialized secretory cells in lactating tissue, high lipid content and composition of the milk, functionally conserved milk proteins (Transferrins/Lactoferrins, Lipocalins and Sphingomyelinase are present in both mammalian and tsetse milk) and transmission of symbiotic bacteria from the mother to the progeny via milk secretion [13]–[15], [65]–[68]. In relation to mammalian lactation and aquaporins, protein and transcript localization studies have been conducted in humans, bovine and mice. Five AQPs (AQP1, AQP3-5, and AQP7) are expressed in the bovine mammary gland [69]. In humans and mice, AQP1 and AQP3 are documented in mammary gland tissues [69]–[71]. In mice, transcripts for AQP4, AQP5, AQP7 and AQP9 are detected in the mammary glands, but the presence of the corresponding proteins has not yet been documented [71]. Currently, no knockdown studies have addressed the functional role of mammalian AQPs during lactation. As mentioned previously, we show that *gmmdripa* and *gmmdripb* are predominately expressed in the milk gland/fat body structure and are critical to fecundity and progeny health. Intrigued by the similarities between tsetse fly and mammalian lactation, we tested the hypothesis that knockdown of AQPs during tsetse lactation could yield milk with increased osmolality due to reduced water content. Indeed combined knockdown of tsetse milk gland AQPs yielded milk with extremely higher that normal osmolality and resulted in progeny receiving less water during nursing, which in turn prompted dehydration and impaired development in the larvae. It is not known if similar phenotypes will occur in mammal progeny when AQP levels are reduced in the mother during lactation. Given the similarities in milk production between tsetse and mammals, negative phenotypes of dehydration and impaired development may also occur in mammalian progeny during periods of obligate nursing following AQP knockdown.

In conclusion, we have identified ten AQPs that are encoded by the *G. morsitans* genome and have characterized their chromosomal distribution and structure/tissue specific expression profiles during blood feeding and pregnancy. Knockdown of *gmmdripa* and *gmmdripb* impedes diuresis significantly with consequences for heat resistance, milk osmolality and impaired juvenile development. The role of *gmmaqp5* remains unknown in relation to tsetse physiology. The results of our study suggest that impairment of AQPs could delay blood digestion, or reduce the movement of water into the milk gland during lactation, representing potential targets to reduce tsetse's overall fitness and reproductive capacity. Also, along with utilization in a control protocol, tsetse AQP regulation and activity could serve as a model that can be easily manipulated for investigating the role of AQPs during lactation.

## Supporting Information

Figure S1 siRNA injection diagram for diuresis/water loss assay, dehydration assay, heat tolerance assay, fecundity and pregnancy length assay, intrauterine larva dehydration assay and milk osmolality assay.
(TIF)

Figure S2 Amino acid alignment of *Glossina morsitans* and *Drosophila melanogaster* aquaporins at the first (A) and second (B) asparagine-proline-alanine (NPA). The NPA domain is highlighted in yellow. (C) Percent amino acid similarity (Bottom) and amino acid differences (Top) between aquaporin proteins.
(TIF)

Figure S3 (A) *gmmdripb* and (B) *gmmdripa in situ* sense hybridization, red, along with milk gland protein (MGP) immunohistochemistry, green, and DAPI staining of nuclei, blue, of a cross section of milk gland tubules. 1 = milk gland lumen; 2 = nuclei; 3 = secretory reservoir.
(TIF)

Table S1 Quantitative and T7-RNAi PCR primers.
(DOCX)

Table S2 Expression of aquaporin (AQP) transcripts during tsetse fly pregnancy. Transcript levels for AQPs were determined by qPCR utilizing the iCycler iQ real-time PCR detection system (Bio-Rad, Hercules). The data were analyzed with software version 3.1 (Bio-Rad). Data represent the mean ± SE for four samples and was normalized to *tubulin*. Figure 3 are those genes that show variation during the course of pregnancy.
(DOCX)

Table S3 Localization of aquaporin (AQP) transcripts in tsetse tissues/structures. Transcript levels for AQPs were determined by qPCR utilizing the iCycler iQ real-time PCR detection system (Bio-Rad, Hercules). The data were analyzed with software version 3.1 (Bio-Rad). Data represent the mean ± SE for four samples and was normalized to *tubulin*. Shown in Figure 3 are those gene significantly higher than tissue average based on ANOVA followed by a Bonferroni corection (P<0.05).
(DOCX)

Table S4 Expression of aquaporin (AQP) transcripts after blood feeding. Transcript levels for AQPs were determined by qPCR utilizing the iCycler iQ real-time PCR detection system (Bio-Rad, Hercules). The data were analyzed with software version 3.1 (Bio-Rad). Data represent the mean ± SE for four samples and was normalized to *tubulin*. Shown in Table 1 is a summary of the time points significantly higher than before blood feeding (0 h) for each gene.
(DOCX)
