# Supplementary figures and images for: Aquaporins Are Critical for Provision of Water during Lactation and Intrauterine Progeny Hydration to Maintain Tsetse Fly Reproductive Success
Source: PLoS Negl Trop Dis. 2014 Apr 24;8(4):e2517. doi: 10.1371/journal.pntd.0002517 (PMC3998938; doi:10.1371/journal.pntd.0002517)

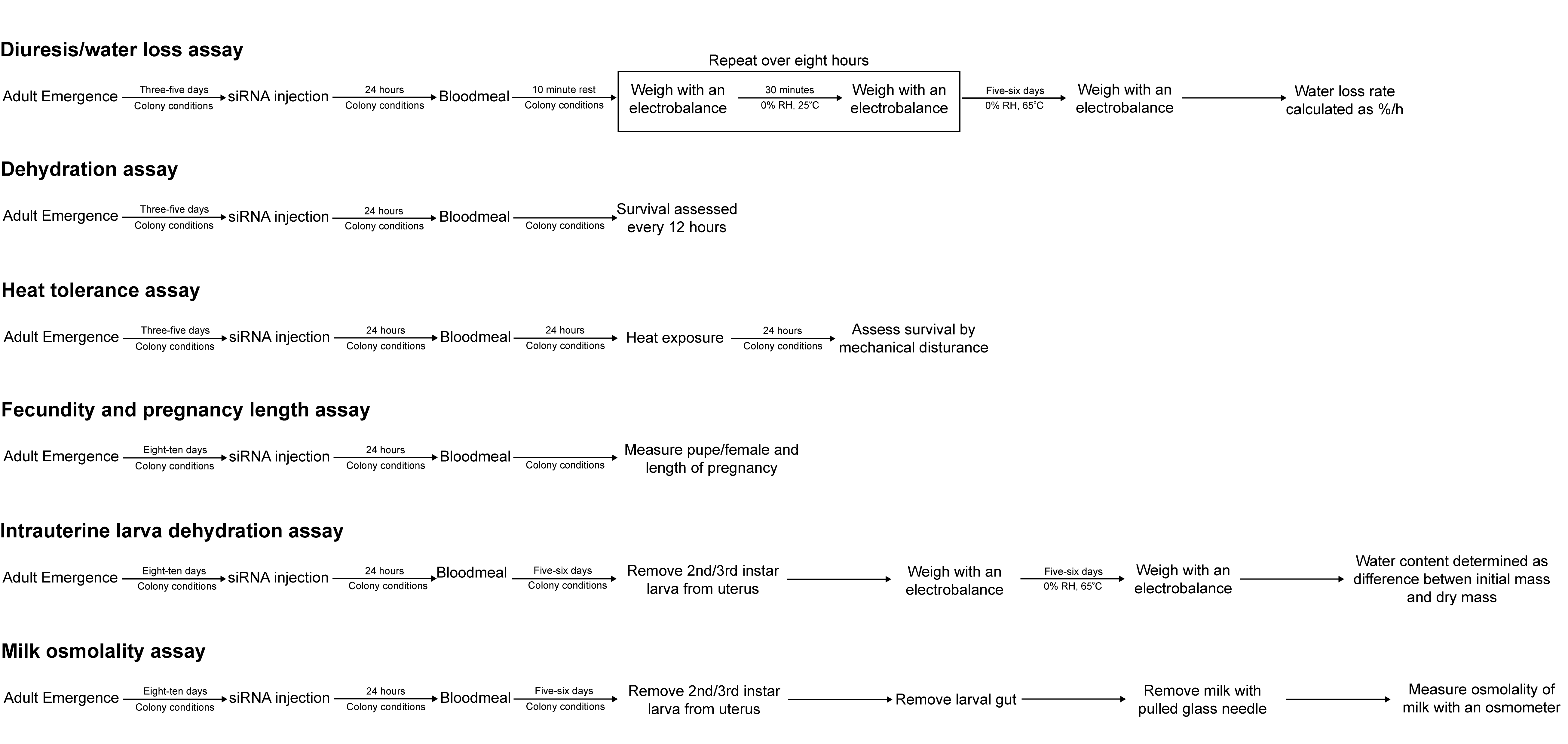

Supplement: Figure S1 — siRNA injection diagram for diuresis/water loss assay, dehydration assay, heat tolerance assay, fecundity and pregnancy length assay, intrauterine larva dehydration assay and milk osmolality assay. (TIF) [file pntd.0002517.s001.tif]

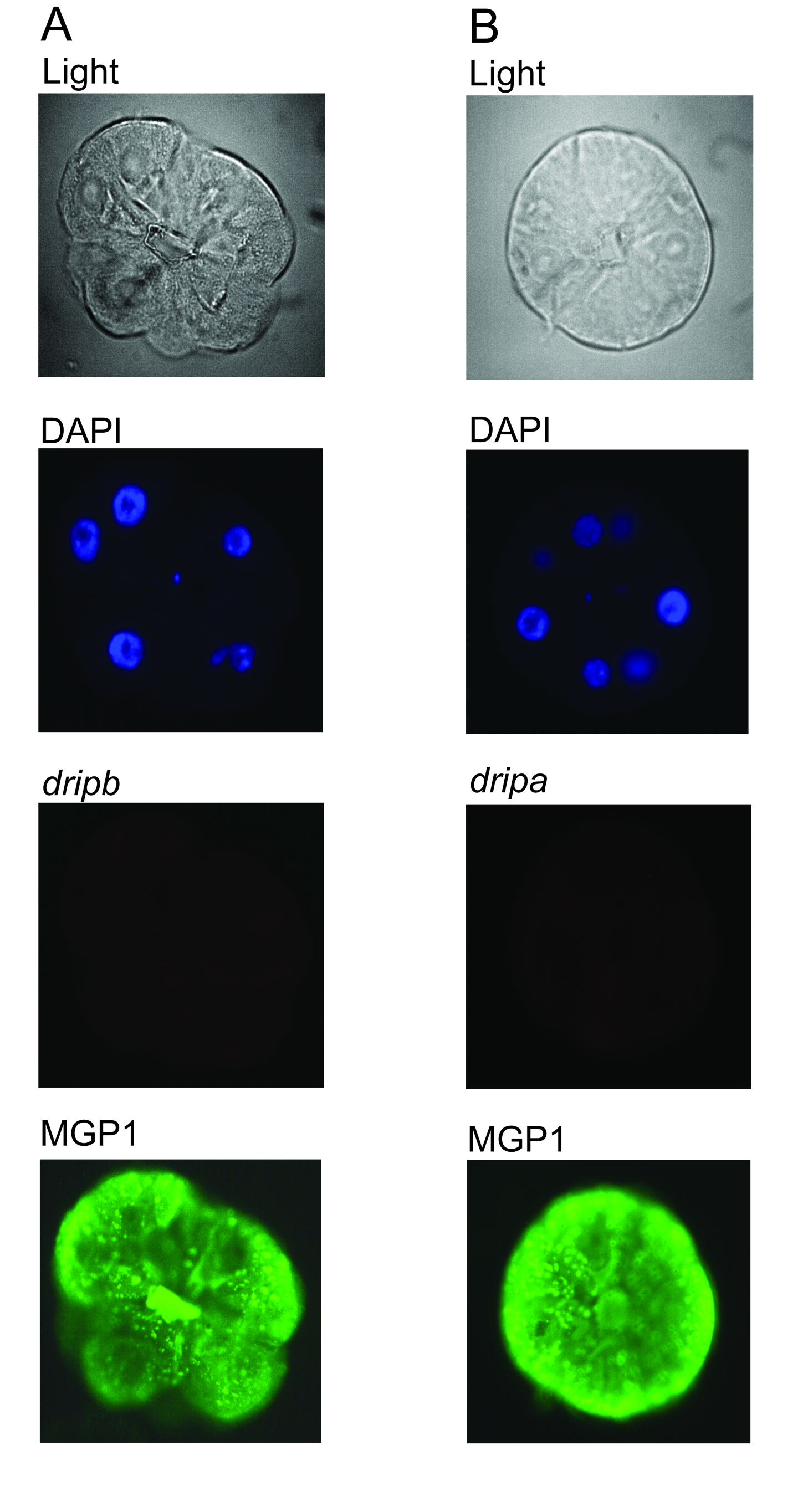

Supplement: Figure S2 — Amino acid alignment of Glossina morsitans and Drosophila melanogaster aquaporins at the first (A) and second (B) asparagine-proline-alanine (NPA). The NPA domain is highlighted in yellow. (C) Percent amino acid similarity (Bottom) and amino acid differences (Top) between aquaporin proteins. (TIF) [file pntd.0002517.s002.tif]

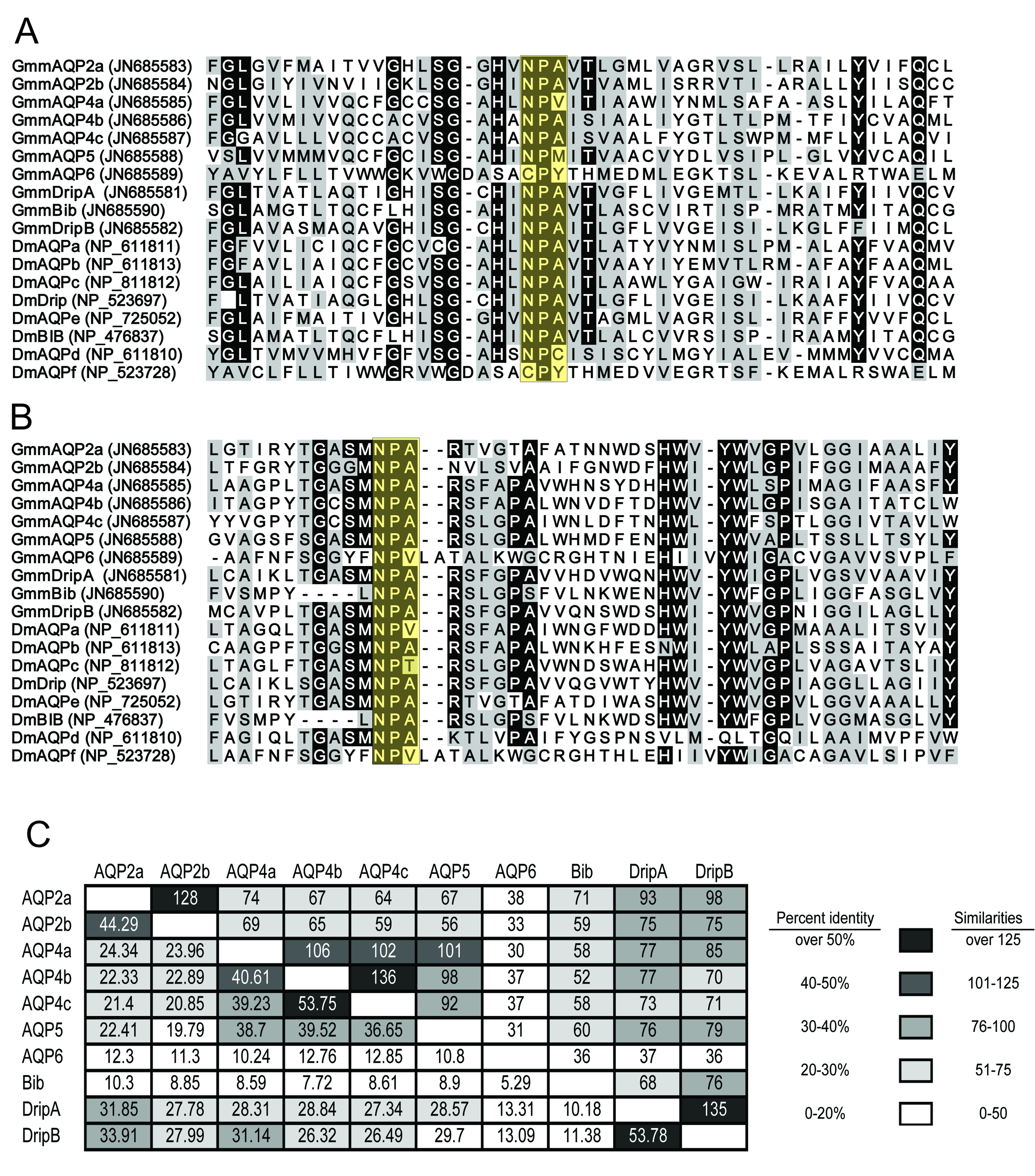

Supplement: Figure S3 — (A) gmmdripb and (B) gmmdripa in situ sense hybridization, red, along with milk gland protein (MGP) immunohistochemistry, green, and DAPI staining of nuclei, blue, of a cross section of milk gland tubules. 1 = milk gland lumen; 2 = nuclei; 3 = secretory reservoir. (TIF) [file pntd.0002517.s003.tif]
