# Supplementary material for: Aquaporins Are Critical for Provision of Water during Lactation and Intrauterine Progeny Hydration to Maintain Tsetse Fly Reproductive Success
Source: PLoS Negl Trop Dis. 2014 Apr 24;8(4):e2517. doi: 10.1371/journal.pntd.0002517 (PMC3998938; doi:10.1371/journal.pntd.0002517)
Supplement: Supplementary file 2 [file pntd.0002517.s004.docx]

qPCR	Forward	Reverse
gmmbib	AATCATAATTCAAGTCGTCTT	TTAATACACGCCGCAACT
gmmdripa	AGCATTCACATTTGGTTT	ATCTCGCCTACTATCAAG
	TGAGCG AGGAAAAGTTAGA	ACATAATAGTACAGTGCGTC
gmmdripb	CATAGTCGGTGTAAAGGAT	CAAGGCAACTGAATAAGC
gmmaqp2a	GCCATTAGGACACTCATC	AATTCAATTCCGACACCTT
gmmaqp2b	AATATCTTCTCCTTGAGCACAT	AATCCGCAATAGCAGAGTT
gmmaqp4a	AATATAACCAATGGCTATCAG	GGCAATAGTAATAACAGGATT
gmmaqp4b	ACAACAGTCCATCCAGAAC	CACACAGCACAGCAAATC
gmmaqp4c	CATTGGTTATATTGGTTCT	TCTCTTCTCTACTCATTG
gmmaqp5	TAGTCGTTATGATAGTAGT	CAATGTTCCATAGATTAGA
gmmaqp6	CCTGGACAATTCTATAAACA	ATATACGCACATGGAAGA
gmmMn/Fe sod	GTTGGTTGGGCTATAACAAG	ATAGTAAGCATGCTCCCAGA
gmmCu/Zn sod	TGAGAAAACCTCGAAAATTG	ATGCCAGTGAGATAAACTCG
gmmCatalase	GATTCAGGTGGGTAAGTTGA	ACAGACGTCCCTGTAACATC
gmmtubulin	CCATTCCCACGTCTTCACTT	GACCATGACGTGGATCACAG
dsRNA		
gmmdripa	TAATACGACTCACTATAGGGAGATTCTTACTGGTTATCATT	TAATACGACTCACTATAGGGAGAGCACTGTACTATTATGTA
gmmdripb	TAATACGACTCACTATAGGGAGAATATCAGTGGTTGTCATA	TAATACGACTCACTATAGGGAGTTATCAAGGCTTCAATA
gmmaqp5	TAATACGACTCACTATAGGGAGACGTTTCTGAATTTATA	TAATACGACTCACTATAGGGAGATTGTAATGGATAGAGTTC
In situ		
gmmdripa	TAATACGACTCACTATAGGGAGATTCTTACTGGTTATCATT	TAATACGACTCACTATAGGGAGAGCACTGTACTATTATGTA
gmmdripb	TAATACGACTCACTATAGGGAGAATATCAGTGGTTGTCATA	TAATACGACTCACTATAGGGAGTTATCAAGGCTTCAATA
gmmaqp5	TAATACGACTCACTATAGGGAGACGTTTCTGAATTTATA	TAATACGACTCACTATAGGGAGATTGTAATGGATAGAGTTC
