# Supplementary material for: Aquaporins Are Critical for Provision of Water during Lactation and Intrauterine Progeny Hydration to Maintain Tsetse Fly Reproductive Success
Source: PLoS Negl Trop Dis. 2014 Apr 24;8(4):e2517. doi: 10.1371/journal.pntd.0002517 (PMC3998938; doi:10.1371/journal.pntd.0002517)
Supplement: Supplementary file 3 [file pntd.0002517.s005.docx]

Days after adult emergence	*gmmbib*	*gmmdripa*	*gmmdripb*	*gmmaqp2a*	*gmmaqp2b*	*gmmaqp4a*	*gmmaqp4b*	*gmmaqp4c*	*gmmaqp5*	*gmmaqp6*
0d	1.294 ± 0.834	1.111 ± 0.079	1.339 ± 0.766	1.082 ± 0.285	1.092 ± 0.781	1.110 ± 0.575	1.114 ± 0.235	0.809 ± 1.745	2.845 ± 0.976	0.932 ± 0.160
5d	1.249 ± 0.219	2.280 ± 0.116	4.187 ± 1.265	1.553 ± 0.680	1.909 ± 1.114	1.659 ± 0.594	2.188 ± 0.752	2.405 ± 2.562	3.769 ± 1.031	1.684 ± 0.566
10d	2.628 ± 0.632	7.125 ± 0.137	6.191 ± 1.884	1.376 ± 0.218	2.454 ± 0.740	3.499 ± 0.727	3.467 ± 0.472	1.921 ± 3.386	5.221 ± 0.972	0.706 ± 0.324
15d	1.065 ± 0.742	6.393 ± 0.261	4.459 ± 0.961	1.497 ± 0.406	1.028 ± 1.320	2.153 ± 0.885	2.862 ± 1.331	3.339 ± 6.317	6.948 ± 1.467	1.212 ± 0.119
20d	1.051 ± 0.315	3.998 ± 0.329	1.839 ± 0.811	0.681 ± 0.741	1.052 ± 1.029	1.425 ± 1.138	2.053 ± 0.949	4.854 ± 11.181	2.690 ± 0.456	1.075 ± 0.159
21d	1.746 ± 1.072	2.663 ± 0.892	1.945 ± 0.646	0.584 ± 0.604	1.658 ± 2.158	1.284 ± 0.844	1.967 ± 1.043	5.053 ± 9.415	2.025 ± 0.612	0.952 ± 0.058
22d	1.934 ± 0.555	8.143 ± 1.161	4.933 ± 0.978	1.709 ± 0.323	2.116 ± 0.956	3.234 ± 1.235	3.264 ± 0.768	5.539 ± 10.859	4.250 ± 1.023	1.651 ± 0.638
25d	1.743 ± 1.143	7.119 ± 2.243	5.746 ± 0.407	1.326 ± 0.388	2.865 ± 1.118	2.905 ± 0.742	1.882 ± 0.876	3.553 ± 2.315	5.415 ± 1.582	1.452 ± 0.345
27d	1.140 ± 1.116	2.925 ± 1.944	5.741 ± 1.234	0.326 ± 0.089	1.465 ± 1.119	1.105 ± 0.643	1.782 ± 0.907	6.553 ± 2.316	2.235 ± 1.083	1.252 ± 0.556
28d	1.379 ± 1.312	3.689 ± 2.245	1.591 ± 0.909	0.326 ± 0.090	1.465 ± 1.120	1.705 ± 0.994	2.111 ± 0.878	4.553 ± 2.317	1.742 ± 1.000	0.893 ± 0.547
