# Supplementary material for: Aquaporins Are Critical for Provision of Water during Lactation and Intrauterine Progeny Hydration to Maintain Tsetse Fly Reproductive Success
Source: PLoS Negl Trop Dis. 2014 Apr 24;8(4):e2517. doi: 10.1371/journal.pntd.0002517 (PMC3998938; doi:10.1371/journal.pntd.0002517)
Supplement: Supplementary file 4 [file pntd.0002517.s006.docx]

Tissue distribution	*gmmbib*	*gmmdripa*	*gmmdripb*	*gmmaqp2a*	*gmmaqp2b*	*gmmaqp4a*	*gmmaqp4b*	*gmmaqp4c*	*gmmaqp5*	*gmmaqp6*
Head	138.708 ± 13.718	0.205 ± 0.145	1.113 ± 0.531	0.400 ± 1.531	0.301 ± 0.552	0.007 ± 1.345	1.445 ± 0.670	1.078 ± 0.670	1.003 ± 0.080	4.723 ± 1.567
Salivary gland	3.438 ± 3.213	127.923 ± 13.718	0.019 ± 0.025	1.783 ± 2.331	0.481 ± 0.388	0.152 ± 0.228	17.869 ± 5.676	2.033 ± 0.574	63.926 ± 0.345	2.856 ± 1.064
Midgut	1.554 ± 1.563	0.316 ± 0.321	0.082 ± 0.140	38.977 ± 6.531	41.305 ± 8.983	6.241 ± 0.412	97.855 ± 21.673	0.832 ± 0.372	0.601 ± 0.089	0.199 ± 0.012
Spermatheca	4.788 ± 4.212	0.131 ± 0.212	0.191 ± 0.972	0.068 ± 2.531	79.043 ± 10.872	0.001 ± 1.234	0.079 ± 0.072	0.798 ± 0.274	0.044 ± 0.011	0.133 ± 0.017
Fat body/Milk gland	1.328 ± 0.987	12.071 ± 1.718	16.026 ± 1.145	0.059 ± 1.931	3.844 ± 2.342	0.012 ± 0.665	0.136 ± 0.036	0.156 ± 0.471	0.123 ± 0.133	0.002 ± 0.003
Ovary/Oocyte	1.046 ± 0.552	0.032 ± 3.718	0.255 ± 0.145	0.058 ± 2.531	97.392 ± 24.174	0.297 ± 0.112	1.002 ± 0.353	0.123 ± 0.273	0.345 ± 0.015	0.004 ± 0.002
Larvae	1.683 ± 0.453	16.432 ± 3.718	0.016 ± 1.145	3.831 ± 3.531	0.022 ± 0.001	0.005 ± 0.087	0.004 ± 0.078	0.387 ± 0.179	0.013 ± 0.023	1.761 ± 0.142
Malphigian tubules	0.486 ± 1.201	11.715 ± 2.718	5.775 ± 0.745	94.241 ± 12.531	0.081 ± 0.012	12.703 ± 2.761	65.552 ± 12.342	0.984 ± 0.370	92.525 ± 13.223	0.026 ± 0.005
Bacteriome	0.040 ± 1.234	0.006 ± 0.718	0.379 ± 1.145	27.019 ± 7.931	0.004 ± 0.196	1.065 ± 0.078	43.427 ± 11.213	0.087 ± 0.853	11.465 ± 3.456	0.006 ± 0.003
Reproductive tract	2.815 ± 2.109	0.001 ± 1.718	0.167 ± 0.745	0.361 ± 2.531	1.271 ± 0.139	0.001 ± 0.243	0.130 ± 0.321	0.001 ± 0.771	0.015 ± 0.193	0.012 ± 0.010
Hindgut	1.496 ± 0.641	0.237 ± 0.234	1.296 ± 2.145	21.296 ± 8.531	0.084 ± 0.026	0.001 ± 0.252	1.745 ± 0.573	2.141 ± 0.871	5.348 ± 0.768	4.108 ± 0.567
