# Supplementary material for: Aquaporins Are Critical for Provision of Water during Lactation and Intrauterine Progeny Hydration to Maintain Tsetse Fly Reproductive Success
Source: PLoS Negl Trop Dis. 2014 Apr 24;8(4):e2517. doi: 10.1371/journal.pntd.0002517 (PMC3998938; doi:10.1371/journal.pntd.0002517)
Supplement: Supplementary file 5 [file pntd.0002517.s007.docx]

Hours after blood feeding	*gmmbib*	*gmmdripa*	*gmmdripb*	*gmmaqp2a*	*gmmaqp2b*	*gmmaqp4a*	*gmmaqp4b*	*gmmaqp4c*	*gmmaqp5*	*gmmaqp6*
0h	1.285 ± 0.468	0.454 ± 0.199	1.545 ± 0.766	0.904 ± 0.285	0.571 ± 0.781	1.227 ± 0.175	0.409 ± 0.235	0.809 ± 1.745	2.845 ± 0.976	0.932 ± 0.160
2h	0.631 ± 0.379	0.360 ± 0.186	2.107 ± 0.647	1.068 ± 0.180	1.909 ± 1.114	1.159 ± 0.594	0.114 ± 0.052	2.405 ± 2.562	4.769 ± 1.327	1.684 ± 0.566
6h	0.604 ± 0.250	0.978 ± 0.207	3.603 ± 0.500	1.376 ± 0.218	2.454 ± 0.740	1.699 ± 0.227	0.267 ± 0.072	1.921 ± 3.386	2.821 ± 0.972	0.706 ± 0.324
12h	4.616 ± 0.542	1.637 ± 0.201	3.577 ± 0.305	1.497 ± 0.106	3.328 ± 1.320	1.785 ± 0.485	1.127 ± 0.331	13.339 ± 6.317	6.948 ± 1.467	1.212 ± 0.119
24h	1.062 ± 0.605	1.665 ± 0.389	3.649 ± 0.918	1.681 ± 0.141	7.552 ± 1.029	4.295 ± 1.138	1.719 ± 0.649	7.854 ± 11.181	4.690 ± 0.456	1.075 ± 0.159
48h	0.640 ± 2.272	1.579 ± 0.032	3.341 ± 0.646	1.584 ± 0.104	9.758 ± 2.158	3.284 ± 0.644	1.267 ± 0.543	5.053 ± 9.415	2.025 ± 2.109	0.952 ± 0.058
72h	0.601 ± 0.695	0.503 ± 0.161	1.491 ± 0.838	0.709 ± 0.323	5.716 ± 0.956	2.120 ± 0.635	0.464 ± 0.768	5.539 ± 10.859	4.250 ± 1.524	0.651 ± 0.138
120h	2.670 ± 1.689	0.459 ± 0.243	1.256 ± 0.407	0.326 ± 0.088	3.465 ± 1.118	1.105 ± 0.242	0.382 ± 0.876	7.553 ± 2.315	2.235 ± 1.582	1.452 ± 0.345
